# B3GNT3 Expression Is a Novel Marker Correlated with Pelvic Lymph Node Metastasis and Poor Clinical Outcome in Early-Stage Cervical Cancer

**DOI:** 10.1371/journal.pone.0144360

**Published:** 2015-12-28

**Authors:** Weijing Zhang, Teng Hou, Chunhao Niu, Libing Song, Yanna Zhang

**Affiliations:** 1 State Key Laboratory of Oncology in South China, Collaborative Innovation Center for Cancer Medicine, Cancer Center, Sun Yat-Sen University, Guangzhou, People’s Republic of China; 2 Department of Urology, Wuhan Union Hospital of Huazhong University of Science and Technology, Wuhan, People’s Republic of China; Centro di Riferimento Oncologico, IRCCS National Cancer Institute, ITALY

## Abstract

**Background:**

The β1,3-N-acetylglucosaminyltransferase-3 gene (*B3GNT3*) encodes a member of the B3GNT family that functions as the backbone structure of dimeric sialyl-Lewis A and is involved in L-selectin ligand biosynthesis, lymphocyte homing and lymphocyte trafficking. B3GNT3 has been implicated as an important element in the development of certain cancers. However, the characteristics of B3GNT3 in the development and progression of cancer remain largely unknown. Thus, our study aimed to investigate the expression pattern and the prognostic value of B3GNT3 in patients with early-stage cervical cancer.

**Methods:**

The mRNA and protein levels of *B3GNT3* expression were examined in eight cervical cancer cell lines and ten paired cervical cancer tumors, using real-time PCR and western blotting, respectively. Immunohistochemistry (IHC) was used to analyze B3GNT3 protein expression in paraffin-embedded tissues from 196 early-stage cervical cancer patients. Statistical analyses were applied to evaluate the association between *B3GNT3* expression scores and clinical parameters, as well as patient survival.

**Results:**

*B3GNT3* expression was significantly upregulated in cervical cancer cell lines and lesions compared with normal cells and adjacent noncancerous cervical tissues. In the 196 cases of tested early-stage cervical cancer samples, the B3GNT3 protein level was positively correlated with high risk TYPES of human papillomavirus (HPV) infection (P = 0.026), FIGO stage (P < 0.001), tumor size (P = 0.025), tumor recurrence (P = 0.004), vital status (P < 0.001), concurrent chemotherapy and radiotherapy (P = 0.016), lymphovascular space involvement (P = 0.003) and most importantly, lymph node metastasis (P = 0.003). Patients with high B3GNT3 expression had a shorter overall survival (OS) and disease-free survival (DFS) compared with those with low expression of this protein. Multivariate analysis suggested that B3GNT3 expression is an independent prognostic indicator for cervical cancer patients.

**Conclusions:**

Our study demonstrated that elevated B3GNT3 expression is associated with pelvic lymph node metastasis and poor outcome in early-stage cervical cancer patients. B3GNT3 may be a novel prognostic marker and therapeutic target for the treatment of cervical cancer.

## Introduction

In women, cervical cancer is the third most commonly diagnosed malignant tumor of the reproductive tract worldwide, accounting for an estimated 274,000 deaths worldwide annually [1]. Numerous studies have indicated that high risk TYPES of human papillomavirus (HPV) infection is a main risk for cervical development, but it remains unsatisfactory in diagnosis and predicting prognosis [2]. Advances in therapeutic methods and diagnostic tools have decreased the incidence and mortality of cervical cancer [3]. However, it is still the major cause of gynecological oncology-related death in developing countries, especially patients with lymph node metastasis [4]. Moreover, lymph node metastasis is the strongest prognostic factor for early-stage cervical cancer (FIGO stage Ib-IIa) and it determines the treatment strategy for cervical cancer [5]. Altered expressions of oncogenes, such as *Bmi-1*, *URG4* and *C14ORF166* [6–8], have been identified as potential prognostic markers in cervical cancer. However, the power of many identified biomarkers to predict lymph node metastasis and clinical outcome of individual tumors is limited. Thus, the identification of novel and specific biomarkers for the early detection and prediction of lymph node metastasis and prognosis in cervical cancer is important.

The gene encoding β1,3-N-acetylglucosaminyltransferase-3 (B3GNT3), formerly called core 1β3GlcNAcT, is located on chromosome 19q13.1 and comprises three exons [9]. It is a member of the β3GlcNAcT family with a full-length mRNA of 2720 bp that encodes a highly conserved 43-kDa protein, which is classified as a type II transmembrane protein [10]. The β3GlcNAcT family comprises at least eight different β3GlcNAcTs that are associated with malignant transformation [11]. Downregulation of B3GNT1 is associated with poor outcome in pancreatic ductal adenocarcinoma [12]. Etcheverry [13] found that B3GNT5 was overexpressed with a hypomethylated promoter in glioblastoma tissues compared with control brain tissues. Shibata [14] proved that B3GNT7, which functions in the biosynthesis of the HMOCC-1 antigen, is expressed in higher in human ovarian cancer cells relative to normal ovaries. The level of B3GNT8 transcript was increased markedly and may be involved in malignancy in leukemia, laryngeal carcinoma, colon and gastric cancer [15, 16, 17, 18].

B3GNT3 expression has been observed in normal tissue, including the colon, jejunum, stomach, esophagus, placenta and trachea [19]. Recently, Ho [20] reported that B3GNT3 might play an important role in suppressing the malignant phenotypes of neuroblastoma cells, including migration and invasion, by suppression of FAK, Akt and ERK, which are important downstream signaling molecules for integrins and numerous growth factor receptors. However, it is reported that B3GNT3 protein participates in the development and progression of human cancers, such as non-Hodgkin lymphoma (NHL), colon cancer, esophageal squamous cell cancer (OSCC), and pancreatic and hepatocellular cancers [10,19,21]. For example, He [21] found an association between the *B3GNT3* locus, CA19-9 levels and the sialyl Lewis A antigen, which might be the potential mechanism in OSCC, pancreatic and hepatocellular cancers. In the etiology of NHL, B3GNT3 plays dominant roles in L-selectin ligand biosynthesis, which is important for tumor cell survival and metastasis [10]. B3GNT3 is likely to be the most probable candidate involved in the biosynthesis of the backbone structure of dimeric sialyl Lewis A (Galβ1–3GlcNAcβ1–3Galβ1–3GlcNAc), which is a cancer associated glycosphingolipid antigen in human colon cancer tissues and the colon cancer cell line Colo205 [19]. Moreover, Yeh et al. [9] reported that B3GNT3 directs the extension of core 1 mucin-type O-glycan, which often forms the 6-sulfo sialyl Lewis x antigen, an epitope expressed in high endothelial venules (HEV) and functions as an L-selectin ligand required for lymphocyte homing. These findings suggested that B3GNT3 may play different roles in the development and progression of various cancers. Nevertheless, studies on the association between B3GNT3 and cancers are rare, and there is no published report on the characteristics of B3GNT3 expression and its clinical/prognostic significance in cervical cancer, particularly the correlation between B3GNT3 expression and pelvic lymph node metastasis (PLNM).

Here, we aimed to explore the expression pattern of B3GNT3 in cervical cancer cell lines and early-stage cervical cancer specimens. Furthermore, we investigate the relationship between it and clinicopathological parameters and analyze its value in prognosis of early-stage cervical cancer patients based on clinical outcome data.

## Methods

### Ethics statement

The research ethics committee of the Cancer Center, Sun Yat-Sen University (China) provided ethical approval for this study, and all patients provided written informed consent. All specimens were handled and stored anonymously according to ethical and legal standards.

### Cell lines

Cervical cancer cell lines were obtained from the American Type Culture Collection (ATCC, MD, USA). The cervical cancer cell lines, including HeLa, HeLa229, HCC94, SiHa, ME-180, MS751, Caski and C-33A, were maintained in RPMI-1640 medium (Gibco BRL, Rockville, MD) supplemented with 10% foetal bovine serum (FBS) (HyClone, Logan, UT, USA) and 1% antibiotics (100 U/ml penicillin and 100 lg/ml streptomycin). Primary normal cervical epithelial cells (NC) were isolated from noncancerous epithelial tissue of uterine cervix and was grown in complete KeratinocyteSFM medium (Invitrogen, Carlsbad, CA, USA). For the use of clinical materials for research purposes, prior written informed consents were obtained from patients and approval were obtained from the Sun Yat-sen University Cancer Center Institutional Research Ethics Committee.

### Samples and clinical characteristics

All patients gave informed written consent for analysis of their tissue for for research purposes, and the study was approved by the Sun Yat-sen University Cancer Center Institutional Review Board. A total of 193 paraffinembedded cervical cancer samples were obtained from the department of Pathology at the Sun Yat-sen University Cancer Center between 2007 and 2009. Tumor staging and clinicopathological classification were performed according to the International Federation of Obstetrics and Gynecology, 2009 (FIGO) classification of carcinoma of the uterine cervix. The 193 patients were in IB1–IIA2 stage cervical cancer and all received a radical hysterectomy and lymphadenectomy without preoperative radiotherapy, chemotherapy, or hormonal therapy. In our study, Patients with any of high-risk factors received postoperative chemotherapy and/or radiotherapy. In detail, “high-risk” factors included PLNM, high differentiation grade, positive parametrial involvement, positive lymphovascular space involvement, positive surgical margin, deep stromal invasion and large tumor size (>4 cm). Besides, patients with only deep stromal invasion or positive surgical margins received radiotherapy. Patients with only lymphovascular space involvement, high differentiation grade, or large tumor size (> 4 cm) received chemotherapy. Clinicopathological data were collected from impatient medical records and summarized in Table 1. All the patients had follow-up records for more than 5 years and the follow-up deadline was January 2014. Survival time was counted from the date of surgery to the follow-up deadline, or the date of death (usually the result of cancer recurrence or metastasis). The median follow-up time for the primary cervical cancer cohort was 51 months, with a follow-up time range of 3 to 99 months. The pre-operative HPV test our hospital used to examine the infection status of patients was polymerase chain reaction detection of HPV. In this cohort, 153 cervical cancer patients showed HPV infection(+), while 40 cervical cancer patients were HPV infection(-). Sixteen fresh cervical cancer and paired adjacent noncancerous cervical tissues (ANT) were collected for real-time PCR, Western blotting and immunohistochemistry (IHC) analysis.

**Table 1:** Clinicopathological features and tumor expression of *B3GNT3* in patients with early-stage cervical cancer.

	Number of cases (%)
**Age (years)**	
≤ 46	100 (51.8)
> 46	93 (48.2)
**FIGO stage**	
Ib1	85 (44.1)
Ib2	29 (15.0)
IIa1	56 (29.0)
IIa2	23 (11.9)
**Histological type**	
Squamous carcinoma	183 (94.8)
Adenocarcinoma	10 (5.2)
**Tumor size (cm)**	
< 4	145 (75.1)
≥ 4	48 (24.9)
**Squamous cell carcinoma antigen concentration (ng/ml)**	
≤ 1.5	94 (48.7)
> 1.5	99 (51.3)
**HPV Infection**	
No	40 (20.7)
Yes	153 (79.3)
**Pelvic lymph node metastasis**	
No	139 (72.0)
Yes	54 (28.0)
**Tumor recurrence**	
No	175 (90.7)
Yes	18 (9.3)
**Vital status (at last follow-up)**	
Alive	158 (81.9)
Dead	35 (18.1)
**Differentiation grade**	
G1	62 (32.1)
G2	115 (59.6)
G3	16 (8.3)
**Extent of myometrial invasion**	
< 1/2	70 (36.3)
≥ 1/2	123 (63.7)
**Positive surgical margin**	
No	180 (93.3)
Yes	13 (6.7)
**Infiltration of parauterine organs**	
No	183 (94.8)
Yes	10 (5.2)
**Lymphovascular space involvement**	
No	166 (86.0)
Yes	27 (14.0)
**Treatment with chemotherapy**	
No	89 (46.1)
Yes	104 (53.9)
**Treatment with radiotherapy**	
No	186 (96.4)
Yes	7 (3.6)
**Treatment with combined chemotherapy and radiotherapy**	
No	177 (91.7)
Yes	16 (8.3)
**Expression of B3GNT3**	
Zero or low	115 (59.6)
High	78 (40.4)

### qPCR

Total cellular RNA was isolated from cultured cells and sixteen fresh cervical cancer and paired adjacent noncancerous cervical tissue samples using the TRIzol Reagent (Invitrogen, Carlsbad, CA, USA) as the manufacturer instructed. These RNA samples were pretreated with RNase-free DNase, and 2μg RNA from each sample was used for complementary deoxyribonucleic acid(cDNA) synthesis priming with random hexamers. The primers were designed by Primer Express v 2.0 software (Applied Biosystems). The following primers were used: B3GNT3, 5′-GCCGGAGATACTTCATCCTG-3′ and 5′-GAGAGGAGCAGGTAGAGGGG-3′; glyceraldehyde-3-phosphate dehydrogenase (GAPDH) gene, 5′-AATGAAGGGGTCATTGATGG-3′ and 5′-AAGGTGAAGGTCGGAGTCAA-3′. The detection reagent used in this experiment was SYBR Green. Total volume of each sample was 10ul: 2 * SYBR Green mix: 5ul; cDNA: 1ul; Forward primer: 0.5ul; Reverse primer 0.5ul; ddH2O 3ul. For PCR-mediated amplification of B3GNT3 cDNA, an initial amplification using B3GNT3-specific primers was performed with a denaturation step at 95°C for 10 min followed by 30 denaturation cycles at 95°C for 60 s, primer annealing at 55°C for 30 s, and primer extension at 72°C for 30 s. Upon the completion of the cycling steps, a final extension step at 72°C for 5 min was done before the reaction mixture was stored at 4°C. Real-time PCR was then employed to determine the fold of increase of B3GNT3 mRNA in cervical cancer cell lines relative to normal cervical epithelial cells and in each of the primary cervical tumors relative to the adjacent normal tissue taken from the same patient. Each experiment was performed in triplicate and repeated at least three times. The mRNA expression of B3GNT3 was normalized to GAPDH.

### Western blotting

Total protein was prepared using the cell total protein extraction kits according to the manufacturer’s instruction (Millipore, Bedford, MA). Equal amounts of each protein extract (30 μg) were electrophoretically separated on 9.5% SDS polyacrylamide gels. Following transfer to polyvinylidene fluoride (PVDF) membranes (Immobilon P, Millipore, Bedford, MA), membranes were blocked for 1 h at room temperature with 5% fat-free milk in Tris-buffered saline containing 0.1% Tween-20 (TBST). The membranes were then incubated with anti-B3GNT3 antibody (1:1000, Proteintech, 18098-1-AP) overnight at 4°C. Horseradish peroxidase-conjugated anti-rabbit IgG antibody (1:2000, Santa Cruz, SC-2004) was used to determine B3GNT3 expression. Finally, B3GNT3 expression was detected using ECL prime Western blotting detection reagent (Amersham) according to the manufacturer’s instructions. Blotted membranes were stripped and re-probed with an anti-Alpha-Tubulin antibody (Sigma, Saint Louis, MO) as a loading control.

### Immunohistochemical(IHC) analysis

Immunohistochemical staining was carried out to assess the protein expression pattern of B3GNT3 in 193 paraffin-embedded human cervical cancer tissues. The procedures were performed with classical protocols. In brief, the paraffin-embedded tissue blocks from cervical cancer patients were cut into 4-μm-thick sections and baked at 65° for 30 min. The sections were deparaffinized with xylene and followed by rehydrated in the water. The slides were submerged into EDTA antigenic retrieval buffer and then microwaving was processed for antigen retrieval. Next, endogenous peroxidase activity was blocked by 3% hydrogen peroxide for 15 min, followed by incubation with 1% bovine serum albumin to block the nonspecific binding. The specimens was incubated overnight at 4°C with a rabbit polyclonal antibody against B3GNT3 (Upstate Biotechnology, 1:100, Proteintech, 18098-1-AP). For negative controls, the anti-B3GNT3 antibody was replaced with normal goat serum. After washing with PBST, the tissue slides were sequentially incubated with a biotinylated anti-rabbit secondary antibody (Sigma) at room temperature for 30 min, followed by further incubation with streptavidin-horseradish peroxidase complex (Sigma) at room temperature for 30 min. The tissue sections were immersed in 3-amino-9- ethyl carbazole and counterstained with 10% Mayer’s hematoxylin, dehydrated and mounted in Crystal Mount.

The evaluation of the degree of immunostaining was reviewed and scored independently by two pathologists blinded to the histopathological features and patient data of the samples. For semi-quantitative analysis, the score of each tissue specimen was based on both the proportion of positively stained tumor cells and the intensity of staining. The immunostaining results for B3GNT3 were scored based on the following criteria: (i) the proportion of positive tumor cells in the tumor tissue: 0 (0%), 1 (1%–10%), 2 (11%–50%), 3 (51%–75%) and 4 (76%–100%); (ii) staining intensity: 0 (no staining), 1 (weak staining = light yellow), 2 (moderate staining = yellow brown), 3 (strong staining = brown). The staining index was calculated as the product of the proportion of positive cells × the staining intensity score (range from 0 to 12). Cutoff values for B3GNT3 expression were chosen on the basis of a measure of heterogeneity with the log-rank test statistical analysis with respect to overall survival. An optimal cutoff value was identified as follows: the staining index score of≥ 6 was considered as tumors with high B3GNT3 expression and ≤4 was considered as tumors with low expression of B3GNT3.

### Statistical analysis

All statistical analyses were performed by using the SPSS 16.0 statistical software package (SPSS, Chicago, USA). Chi-square test and Fisher’s exact test were applied to assess the relationship between B3GNT3 expression and clinicopathological characteristics. Bivariate correlations between variables were calculated by Spearman’s rank correlation coefficients. Survival curves were plotted by the Kaplan-Meier method, and differences were analyzed using the log-rank test. A Cox regression proportional hazards model was used for univariate and multivariate analyses to determine the independent significance of relevant clinical covariates. Values of *P < 0*.*05* in all cases were considered statistically significant.

## Results

### Elevated expression of B3GNT3 in cervical cancer cell lines

Real-time PCR and western blotting were used to examine levels of B3GNT3 expression in eight cervical cancer cell lines (HeLa, HeLa 229, HCC 94, C33A, CaSki, MS751, ME-180 and SiHa) compared with a normal cervical epithelial cell line (NC).

**Fig 1:**
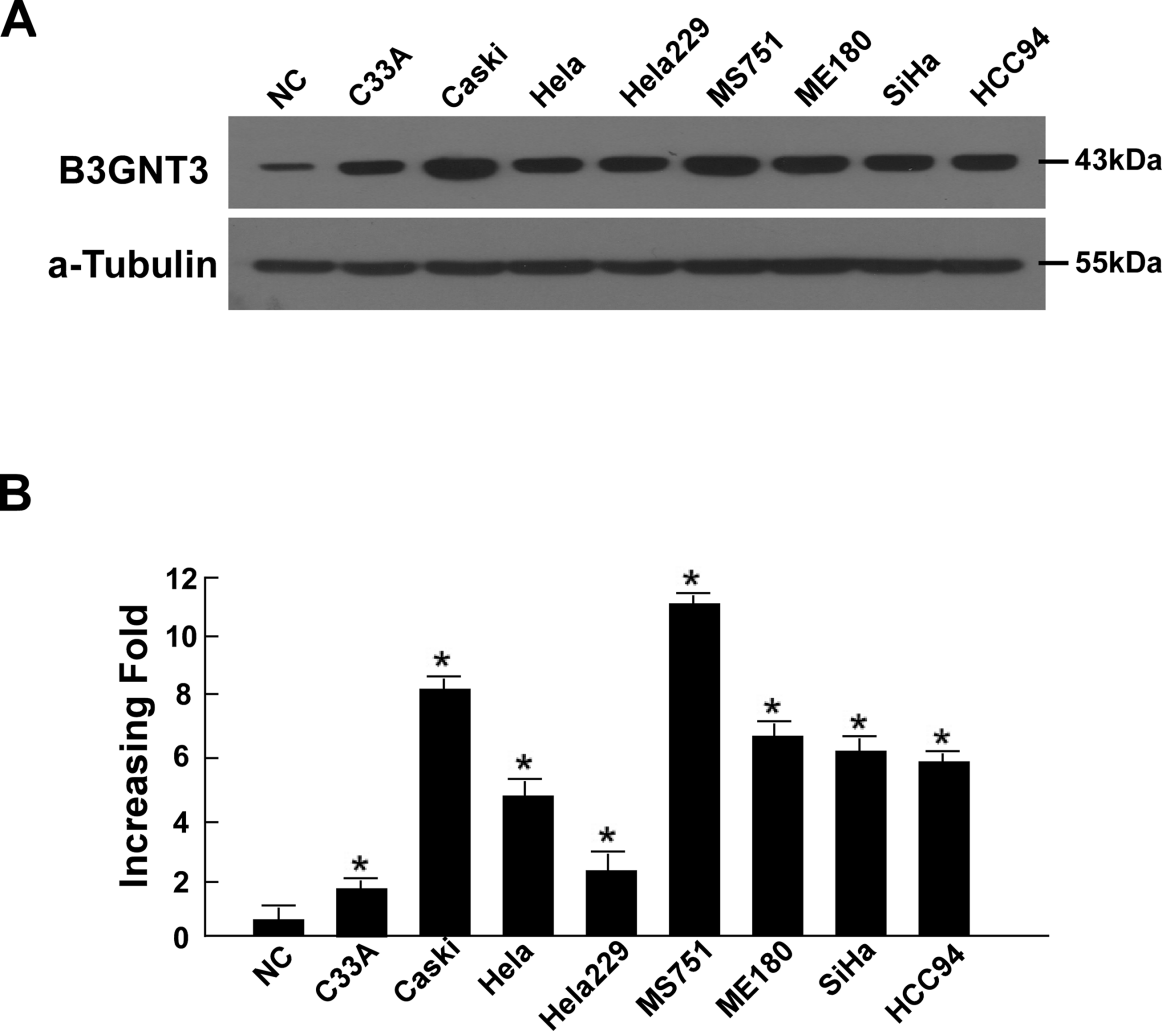
Overexpression of B3GNT3 mRNA and protein in cervical cancer cell lines. (a and b) Expression of B3GNT3 mRNA and protein in cervical cancer cell lines (C33a, Ca Ski, HeLa, HeLa 229, MS751, ME-180, SiHa and HCC 94) and normal cervical cell lines were examined by western blotting (a) and quantitative real-time PCR (qPCR) (b). The expression levels were normalized against α-tubulin and GAPDH, respectively. The error bars represent the standard deviation of the mean (SD) calculated from three parallel experiments. *P < 0.05.

Western blotting analysis showed that all eight cervical cancer cell lines (MS751, C33A, Hela, HeLa229, SiHa, HCC94, CaSki, and ME-180) exhibited significantly higher levels of B3GNT3 protein compared with NC (Fig 1A). *B3GNT3* mRNA expression was at least 3.5-fold greater in the cervical cancer cell lines compared with NC (Fig 1B). These results demonstrated that B3GNT3 is upregulated in cervical cancer cell lines.

### B3GNT3 is overexpressed in cervical cancer tissues

**Fig 2:**
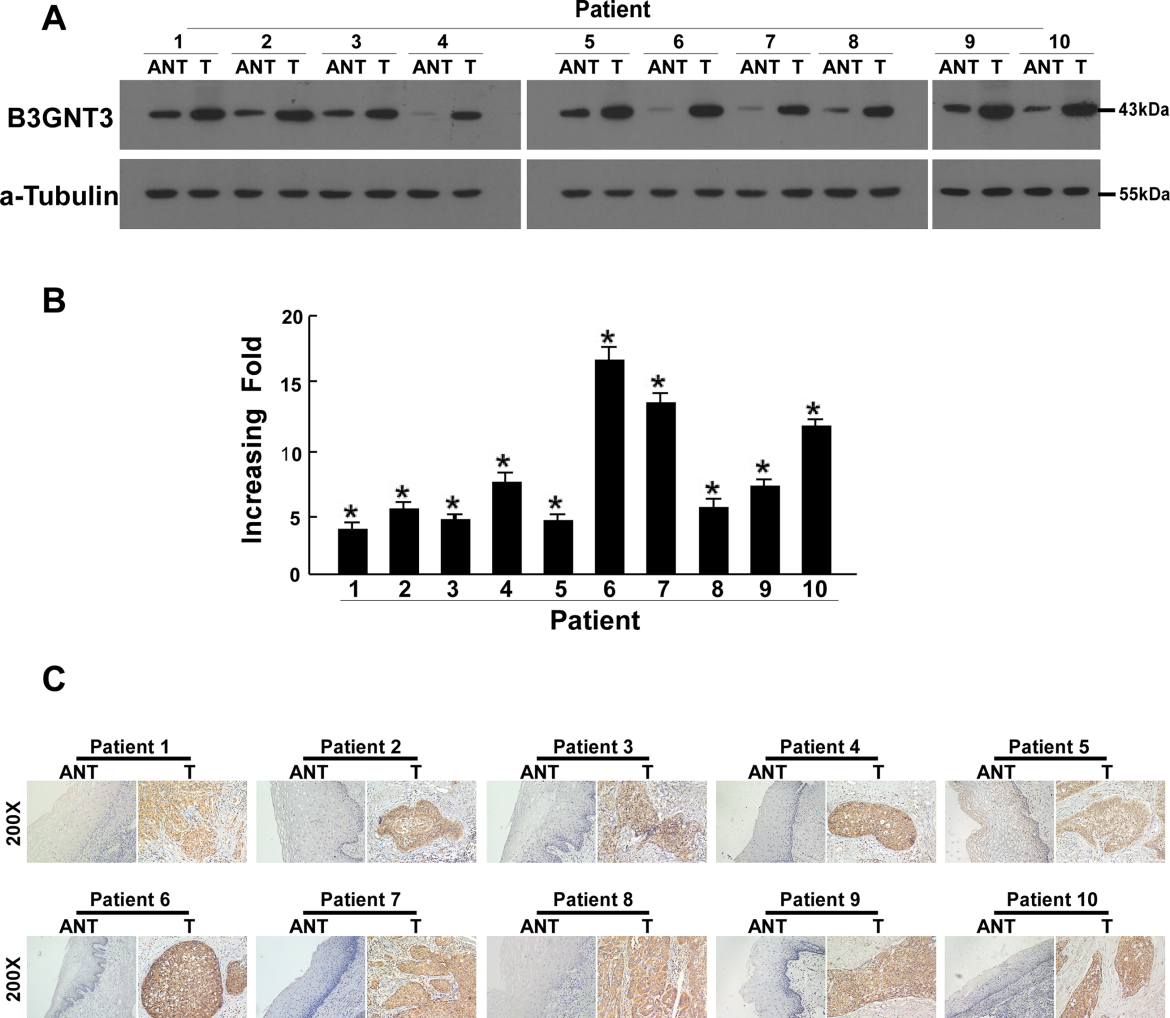
Overexpression of B3GNT3 mRNA and protein in cervical cancer tissues. (a) Representative images of western blotting analyses of the B3GNT3 protein expression in ten matched pairs of cervical cancer tissues (T) and adjacent noncancerous tissues (ANT). α-Tubulin was used as the loading control. (b) The average T/ANT ratios of *B3GNT3* mRNA expression in the paired cervical cancer (T) and adjacent noncancerous tissues (ANT) were quantified by quantitative real-time PCR (qPCR) and normalized against GAPDH. The error bars represent the standard deviation of the mean (SD) calculated from three parallel experiments. (c) Immunohistochemical assay of B3GNT3 protein expression in ten pairs of matched cervical cancer tissues. *P < 0.05.

After that, whether the B3GNT3 upregulation found in cervical cancer cell lines was clinically relevant was confirmed by quantitative real-time PCR (qPCR) and western blotting analysis in 10 cervical cancer tissues (T) matched with adjacent noncancerous tissue samples (ANT). As shown in Fig 2B, the expression of *B3GNT3* mRNA was dramatically higher in the 10 cervical cancer tissue specimens than in the paired normal tissues, with the differential expression level ranging from 4.2- to 17.4-fold. Consistent with these results, the B3GNT3 protein showed higher abundance in all 10 cervical cancer samples compared with the matched ANT (Fig 2A).

### Association between B3GNT3 overexpression and clinical features of cervical cancer

**Fig 3:**
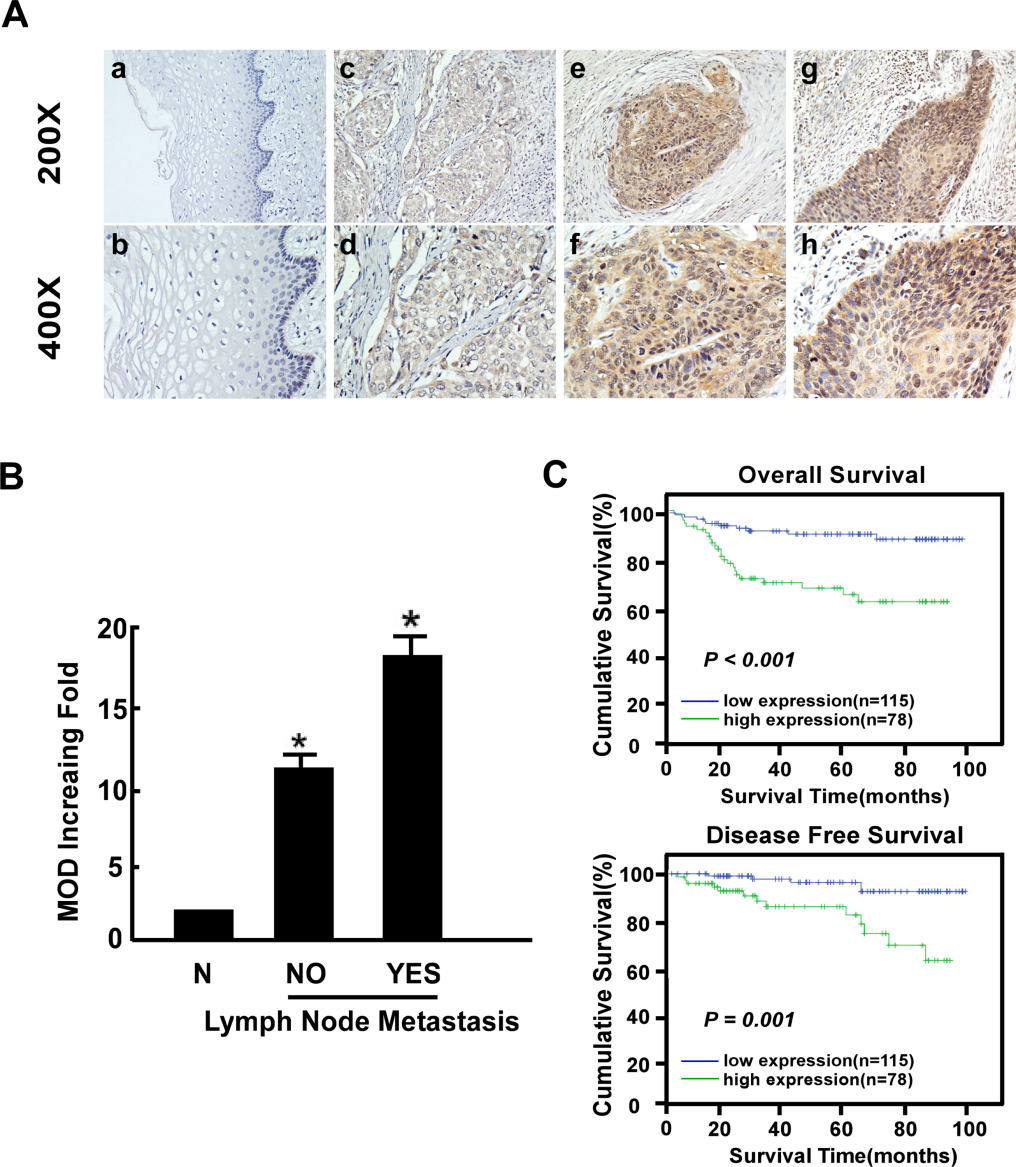
Immunohistochemical detection of B3GNT3 protein expression in paraffin-embedded tissues. Positive B3GNT3 staining was observed mainly in the cytoplasm of cervical cancer cells. (A) a and b, B3GNT3 expression was not detected in normal cervical tissues; c and d, representative images of weak B3GNT3 staining in cervical cancer tissues; e and f, representative images of moderate B3GNT3 staining in cervical cancer tissues; g and h, representative images of strong B3GNT3 staining in cervical cancer tissues. (B) The statistical analyses of the average mean optical density (MOD) of B3GNT3 staining in the lymph node metastasis group and the lymph node metastasis-free group. *P < 0.05. (C) Kaplan-Meier curves of univariate analysis data (log-rank test).The overall survival (OS) and disease-free survival (DFS) for the patients with high versus low B3GNT3 expression.

Immunohistochemistry (IHC) was performed to investigate the expression pattern of B3GNT3 protein in a retrospective cohort of 193 cervical cancer cases, including 85 cases of stage IB1 (44.1%), 29 cases of stage IB2 (15.0%), 56 cases of stage IIA1 (29.0%) and 23 cases of stage IIA2 (11.9%). As shown in Fig 3A, the immunoreactivity of B3GNT3 protein was detected at variable levels and localized mainly in the cellular plasma. Positive staining for B3GNT3 protein was seen in 78 cases (40.4%) of the 193 enrolled patient samples, while 115 cases (59.6%) stained negatively or only weakly for B3GNT3 protein (Table 1). Intense expression of B3GNT3 protein was noted in 24.7% (21/85), 41.4% (12/29), 58.9% (33/56), and 52.2% (12/23) of cervical cancer of FIGO stage Ib1, Ib2, IIa1and IIa2, respectively (*P* < 0.05, χ^2^ test). Interestingly, quantitative IHC staining data revealed that the mean optical density (MOD) values of B3GNT3 staining in the lymph node metastasis group were statistically higher than that in the lymph node metastasis-free group (P < 0.001, Fig 3B).

IHC staining of B3GNT3 protein expression levels was analyzed further to determine their relationship with the clinicopathological characteristics of cervical cancer. As summarized in Table 2, high B3GNT3 protein expression tend to be strongly correlated with HPV infection, FIGO stage, tumor size, tumor recurrence, vital status, concurrent chemotherapy and radiotherapy, lymphovascular space involvement and most importantly, lymph node metastasis (Table 2). These data were further confirmed by Spearman’s correlation analysis (Table 3). However, no significant relationships were found between B3GNT3 protein expression and patient age, histological differentiation grade, squamous cell carcinoma antigen, histological type, chemotherapy, radiation, myometrium invasion, properties of the surgical margin and infiltration of parauterine organ in the patients with cervical cancer.

**Table 2:** Correlation between *B3GNT3* expression and the clinicopathological features of early-stage cervical carcinoma.

	Total	B3GNT3 Zero or weak expression (%)	B3GNT3 Moderate or strong expression (%)	P value from Chi-squared test	P value from Fisher’s exact test
**Age (years)**				0.316	0.379
≤ 46	100	63 (32.7)	37 (19.2)		
> 46	93	52 (26.9)	41 (21.2)		
**Histological type**				0.195	0.207
Adeno cell carcinoma	10	4 (2.1)	6 (3.1)		
Squamous cell carcinoma	183	111 (57.5)	72 (37.3)		
**HPV infection**				0.026	0.030
No	40	30 (15.5)	10 (5.2)		
Yes	153	85 (44.1)	68 (35.2)		
**Clinical stage**				<0.001	-
Ib1	85	64 (33.2)	21 (10.9)		
Ib2	29	17 (8.8)	12 (6.2)		
IIa1	56	23 (11.9)	33 (17.1)		
IIa2	23	11 (5.7)	12 (6.2)		
**Pelvic lymph node metastasis**				0.003	0.003
Absent	139	92 (47.7)	47 (24.3)		
Present	54	23 (11.9)	31 (16.1)		
**Squamous cell carcinoma antigen concentration (ng/ml)**				0.143	0.186
≤ 1.5	94	61 (31.6)	33 (17.1)		
> 1.5	99	54 (28.0)	45 (23.3)		
**Tumor size (cm)**				0.025	0.028
< 4	145	93 (48.2)	52 (26.9)		
≥ 4	48	22 (11.4)	26 (13.5)		
**Tumor recurrence**				0.004	0.005
No	175	110 (57.0)	65 (33.7)		
Yes	18	5 (2.6)	13 (6.7)		
**Vital status (at last follow-up)**				<0.001	<0.001
Alive	158	104 (53.9)	54 (28.0)		
Dead	35	11 (5.7)	24 (12.4)		
**Differentiation grade**				0.052	-
G1	62	37 (19.2)	25 (13.0)		
G2	115	64 (33.2)	51 (26.4)		
G3	16	14 (7.2)	2 (1.0)		
**Chemotherapy**				0.673	0.769
No	88	51 (26.4)	37 (19.2)		
Yes	105	64 (33.2)	41 (21.2)		
**Radiotherapy**				0.893	1.000
No	186	111 (57.5)	75 (38.9)		
Yes	7	4 (2.1)	3 (1.5)		
**Combined chemotherapy and radiotherapy**				0.016	0.030
No	177	110 (57.0)	67 (34.7)		
Yes	16	5 (2.6)	11 (5.7)		
**Extent of myometrial invasion**				0.315	0.361
<1/2	70	45 23.3)	25 (12.9)		
≥1/2	123	70 (36.3)	53 (27.5)		
**Positive surgical margin**				0.057	0.078
No	180	104 (53.9)	76 (39.4)		
Yes	13	11 (5.7)	2 (1.0)		
**Infiltration of parauterine organs**				0.491	0.743
No	183	108 (56.0)	75 (38.9)		
Yes	10	7 (3.6)	3 (1.5)		
**Lymphovascular space involvement**				0.003	0.005
No	166	106 (54.9)	60 (31.1)		
Yes	27	9 (4.7)	18 (9.3)		

**Table 3:** Spearman correlation analysis of B3GNT3 against clinicopathological factors.

Variable	B3GNT3 expression	
	Spearman’s correlation coefficient	*P*-value
Age	0.072	0.319
Histological type	-0.093	0.197
HPV infection	0.161	0.026
FIGO Stage	0.287	<0.001
Pelvic lymph node metastasis	0.216	0.003
Squamous cell carcinoma antigen concentration (ng/ml)	0.105	0.145
Tumor size	0.161	0.025
Tumor recurrence	0.208	0.004
Vital status	0.270	<0.001
Differentiation grade	-0.063	0.386
Survival time	-0.214	0.003
Chemotherapy	-0.030	0.674
Radiation	0.010	0.894
Concurrent chemotherapy and radiotherapy	0.174	0.016
Myometrial invasion	0.072	0.318
Positive surgical margin	-0.137	0.057
Infiltration of parauterine organs	-0.050	0.493
Lymphovascular space involvement	0.216	0.003

### High B3GNT3 expression predicts poor prognosis of patients with early-stage cervical cancer

**Fig 4:**
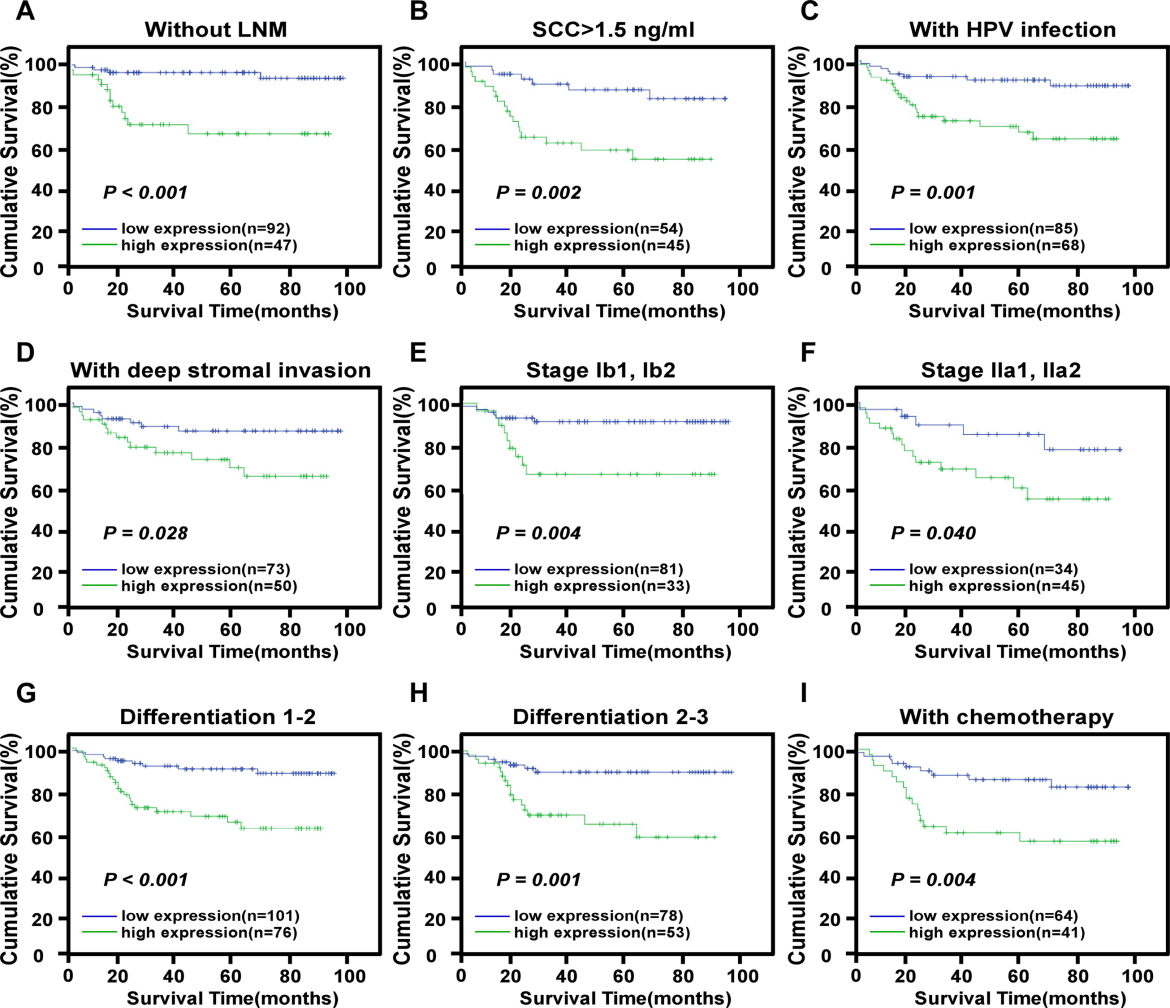
Kaplan-Meier curves of univariate analysis data (log-rank test). (a) The overall survival (OS) of the patients without lymph node metastasis with high versus low B3GNT3 expression. (b) The OS for the patients with squamous cell carcinoma antigen > 1.5ng/ml with high versus low B3GNT3 expression. (c) The OS for the patients with HPV infection with high versus low B3GNT3 expression. (d) The OS for the patients with deep stromal invasion with high versus low B3GNT3 expression. (e) The OS for the patients at stages Ib1-Ib2 with high versus low B3GNT3 expression. (f) The OS for the patients at stages IIa1-IIa2 with high versus low B3GNT3 expression. (g) The OS for the patients at differentiation 1–2 with high versus low B3GNT3 expression. (h) The OS for the patients at differentiation 2–3 with high versus low B3GNT3 expression. (i) The OS for the patients with high versus low B3GNT3 expression who received chemotherapy.

In our study cohort, the prognostic impact of B3GNT3 protein expression in cervical cancer was explored using Kaplan–Meier analysis and the log-rank test. The log-rank test showed that the survival time was significantly different between the low and high B3GNT3 protein expression groups (both P < 0.001, Fig 3C). Patients with high B3GNT3 levels had a significantly shorter overall survival (OS) and disease free survival (DFS) than those with low levels. The cumulative OS and DFS rates were 90.5% and 97.2%, respectively, in the low B3GNT3 group, whereas they were only 65.7% and 84.7%, respectively, in the high B3GNT3 group. A validation cohort was employed to further evaluate the prognostic value of B3GNT3 for specific subgroups of patients, which were stratified according to age, HPV infection, FIGO Stage, PLNM, squamous cell carcinoma antigen, tumor size, differentiation grade, chemotherapy, radiation, concurrent chemotherapy and radiotherapy, myometrium invasion, properties of the surgical margin, infiltration of parauterine organ and lymphovascular space involvement. The expression of B3GNT3 protein was strongly associated with the OS duration of patients without PLNM (log-rank test, P < 0.001), with squamous cell carcinoma antigen ≥ 1.5 ng/ml (log-rank test, P = 0.002), with HPV infection (log-rank test, P = 0.001), with deep stromal invasion (log-rank test, P = 0.028), with clinical stage (Stage Ib1-Ib2, log-rank test, P = 0.004; Stage IIa1-IIa2, log-rank test, P = 0.040), with histological differentiation grade (Grade 1–2, log-rank test, P < 0.001; Grade 2–3, log-rank test, P = 0.001), and with chemotherapy (log-rank test, P = 0.004) (Fig 4). Univariate Cox regression analyses revealed that at a higher level of B3GNT3, PLNM, squamous cell carcinoma antigen, lymphovascular space involvement and recurrence predict a poor prognosis for early-stage cervical cancer patients (Table 4). Then, multivariate analysis using the Cox proportional hazards model for all variables that were significant in the univariate analysis revealed that B3GNT3 expression, PLNM and recurrence served as independent prognostic factors for patients with cervical cancer (Table 4). Thus, our results indicated that B3GNT3 protein may represent a potential prognostic indicator for early-stage cervical cancer patients.

**Table 4:** Univariate and multivariate analyses of prognostic factors in early-stage cervical carcinoma using a Cox-regression model.

	Univariate analysis			Multivariate analysis		
	No. of patients	P value	Regression coefficient (SE)	P value	Relative risk	95% confidence interval
**B3GNT3 expression**		< 0.001	3.754 (0.365)	0.018	2.470	1.165–5.239
Low	115					
High	78					
**Pelvic lymph node metastasis**		< 0.001	3.429 (0.340)	0.029	2.210	1.084–4.505
Absent	139					
Present	54					
**Squamous cell carcinoma antigen concentration (ng/ml)**		0.017	2.443 (0.374)	0.077	1.955	0.929–4.115
≤ 1.5	94					
> 1.5	99					
**Lymphovascular space involvement**		0.038	2.229 (0.387)	0.998	1.001	0.432–2.319
No	166					
Yes	27					
**Recurrence**		< 0.001	4.577 (0.375)	0.004	3.163	1.439–6.953
No	175					
Yes	18					

## Discussion

In the present study, we showed, for the first time, that expression of B3GNT3 protein is upregulated in cervical cancer and correlates with clinical characteristics, especially PLNM of patients with early-stage cervical cancer. Furthermore, high B3GNT3 protein expression reduced the OS and DFS of these patients, particularly those who received chemotherapy. Multivariate analysis revealed that B3GNT3 expression might be an independent prognostic indicator of survival in cervical cancer patients.

B3GNT3 is involved in the biosynthesis of poly-N-acetyllactosamine chains and the biosynthesis of the backbone structure of dimeric sialyl Lewis A. The essential roles of B3GNT3 in tumorigenesis have been revealed recently [10, 19, 20, 21]. Shiraishi [19] demonstrated that B3GNT3 was highly expressed in the colon cancer cell line Colo205. They reported that human colon cancer tissues and the colon cancer cell line Colo205 contain cancer-associated glycosphingolipids with dimeric Lewis A antigens (Galβ1–3(Fucα1–4)GlcNAcβ1–3Galβ1–3(Fucα1–4)GlcNAc) and B3GNT3 is likely to be the most probable candidate involved in the biosynthesis of the backbone structure of dimeric sialyl Lewis A (Galβ1–3GlcNAcβ1–3Galβ1–3GlcNAc). Cerhan [10] found that B3GNT3 is related to immune function and inflammation, and plays an important role in lymphocyte trafficking and migration, which lead to tumor cell survival and metastasis in NHL. Another study indicated that the *B3GNT3* locus is associated with CA19-9 levels in pancreatic cancer [21]. B3GNT3 is a β-1, 3-N-acetylglucosmaniyltransferase involved in production of the backbone structure of dimeric sialyl Lewis A, which is the carbohydrate antigenic epitope of CA19-9. Therefore, this might be the potential mechanism underlying the association between variants in the *B3GNT3* locus and CA19-9 concentrations. Their study [21] suggested that B3GNT3 plays an oncogenic role in certain types of cancer. However, Ho [20] reported that B3GNT3 might play a critical role in suppressing the malignant properties of neuroblastomas and its altered expression might contribute to the pathogenesis of neuroblastoma. It is common that certain genes have oncogenic roles in several types of cancers but have anti-oncogenic roles in other types of cancers. For example, PTK6 is overexpressed in invasive ductal breast carcinoma, serous ovarian carcinoma, non-small cell lung cancer, colon carcinoma and head and neck cancers [22–26]. In contrast, PTK6 may be an important tumor suppressor in various human cancers, such as human laryngeal squamous cell carcinoma and esophageal squamous cell carcinoma [27, 28]. Besides, the role of B3GNT3in cervical carcinoma is unclear. Therefore, we assessed whether B3GNT3 is upregulated in cervical cancer and is clinically associated with the development and progression of cervical cancer. In our study, we reported that the B3GNT3 mRNA and protein were overexpressed in cervical cancer cell lines and cervical cancer samples (stages Ib-IIa). A commercially available antibody against B3GNT3 was used in immunohistochemistry. IHC staining showed negatively or only weakly in normal cervical tissue. However, positive staining for B3GNT3 protein was seen in cervical cancer specimens. Moreover, our results of Realtime-PCR, Western blotting and IHC were consistent. Therefore, this polyclonal antibody specifically recognizes only B3GNT3 in cervical cancer tissues. Furthermore, we found that B3GNT3 expression correlates with clinical characteristics of patients with early-stage cervical cancer such as HPV infection, FIGO stage, tumor size, tumor recurrence, vital status, concurrent chemotherapy and radiotherapy, lymphovascular space involvement and most importantly, lymph node metastasis. Our results strongly indicated that this protein plays an oncogenic role in cervical cancer and may represent a biomarker for the identification of subsets of cervical cancer patients with a more aggressive form of the disease. Univariate and multivariate analysis revealed that B3GNT3 expression might be an independent prognostic indicator of survival in early-stage cervical cancer patients. Our results suggested the important role of B3GNT3 protein in the prognosis of patients with early-stage cervical cancer.

Classically, LNM is the key to determining the treatment and prognosis of early-stage cervical cancer [29, 30]. Thus far, radical hysterectomy plus lymphadenectomy or chemoradiation have remained the standard treatment for early-stage cervical cancer patients [31, 32]. For patients with LNM, chemoradiation is required, which would make the initial surgical procedure unnecessary in retrospect. Thus, preoperative prediction of LNM is very important [33, 34]. However, no accurate, preoperative marker has been established to predict LNM. Such a marker would enable better decision making for treatment, avoid unnecessary surgical intervention, and reduce morbidity. In our cohort, we found that B3GNT3 protein expression strongly correlated with PLNM. Multivariate analysis demonstrated that PLNM is predictive of the overall survival (OS) of early-stage cervical cancer patients (Table 4). In addition, we observed a correlation between OS and high B3GNT3 protein expression in the “without lymph node metastasis” and “with lymph node metastasis” subgroups. Finally, we found a significant association between shorter OS and high B3GNT3 protein expression in the “without lymph node metastasis” subgroup, which suggested that B3GNT3 might be a useful marker to predict poor overall survival of cervical cancer patients without LNM. Nevertheless, further study extended to a larger cohort of patients with LNM, and further investigation of the mechanism by which B3GNT3 is involved in the LNM of cervical cancer, are required.

Further treatments are required for patients with high risk factors. Radical hysterectomy (RH, hysterectomy plus pelvic lymph node dissection) or chemoradiation are widely used treatments for early-stage (Ib to IIa) cervical cancer, as well as for stage IIb disease, in some European and Asian countries [35]. Adjuvant treatments after RH are still controversial. Yamashita [36] found that survival results with concurrent chemoradiation therapy (CCRT) and with conventional surgery plus postoperative radiotherapy (PORT) for patients with stage IIB cervical carcinoma are comparable. In the high-risk group, postoperative CCRT showed better prognosis of FIGO stage IB1-IIB cervical cancer patients compared with postoperative radiotherapy alone [37]. An intergroup study, conducted by the GOG, RTOG, and Southwest Oncology Group, compared PORT alone with postoperative CCRT and indicated that overall and progression-free survivals were superior for patients receiving CCRT [38]. Li [39] suggested that cervical cancer patients treated with chemotherapy, especially those with early-stage disease, had increased 5-year OS and DFS rates compared with the radiotherapy group. In the present study, “high-risk” prognostic factors included PLNM, high differentiation grade, positive parametrial involvement, positive lymphovascular space involvement, positive surgical margin, deep stromal invasion and large tumor size (>4 cm). Patients with any of these high-risk factors received postoperative chemotherapy and/or radiotherapy. Besides, patients with only deep stromal invasion or positive surgical margins received radiotherapy. Patients with only lymphovascular space involvement, high differentiation grade, or large tumor size (> 4 cm) received chemotherapy. Interestingly, our results showed that expression levels of B3GNT3 protein are correlated with the concurrent chemotherapy and radiotherapy subgroup, while there was no association between B3GNT3 expression and the postoperative chemotherapy subgroup or postoperative radiotherapy subgroup. Moreover, we found that B3GNT3 overexpression was associated with a significantly shorter OS in the chemotherapy subgroup, whereas no correlation was observed in the subgroup receiving only radiotherapy or concurrent chemotherapy and radiotherapy. Thus, our results demonstrated that testing B3GNT3 may be a useful marker for predicting the prognosis of early-stage cervical cancer patients who require chemotherapy.

Persistent HPV infections lead to most of the more than 500 000 cases of cervical carcinoma every year worldwide [40]. To date, more than 200 HPV types have been reported and HPV16 is by far the most carcinogenic in terms of numbers of cases of cervical cancer [41]. The time from infection to invasive cancer is shorter for HPV16 than for other HPV types, but the determinants of invasion other than HPV type are unknown [42]. So far, studies have indicated that oncogenes and microRNAs play significant roles in the manifestation of HPV infections in cervical cancer [43, 44, 45]. In our study, the positive rate of high-risk HPV infection was 79.3% (153/193) in cervical cancer, according to the HPV-DNA chip results from clinical data. We indicated that expression levels of B3GNT3 correlated with HPV infection, which was proved by Chi-squared test, Fisher’s exact test and Spearman’s correlation analysis. Higher levels of B3GNT3 protein expression were observed in HPV-positive cervical cancer tissues (44.4%), whereas HPV-negative cases expressed less B3GNT3 (25%). Hence, the overexpression of B3GNT3 in the HPV-positive cervical cancer tissues prompted us to hypothesize that B3GNT3 might make the cervix more prone to HPV infection, which will cause invasion and metastasis of cervical cancer. However, the mechanism was not determined in our study and further exploration is required in the future.

The present study demonstrated that B3GNT3 is an oncogene in early-stage cervical cancer and further exploration of the mechanism by which B3GNT3 is involved in the progression and LNM of cervical cancer is required. B3GNT3 plays an important role in lymphocyte homing, lymphocyte trafficking and L-selectin ligand biosynthesis [9]. Accumulating evidence suggested that tumor cell migration and metastasis may share similar mechanisms with lymphocyte trafficking [46], which support our results that the upregulation of B3GNT3 may cause lymph node metastasis. Moreover, L-selectin was detected in both primary and metastatic tumors, and it facilitated tumor cells adherence to lymph nodes in oral and pharyngeal squamous cell carcinoma [47]. Dallas [48] showed that L-selectin plays a pivotal role in cancer metastasis by interacting with endothelial L-selectin ligands induced proximal to established tumor cell emboli. Our study indicated that B3GNT3 overexpression might be significantly associated with LNM. Thus, we hypothesized that B3GNT3 might facilitate metastasis via modulating L-selectin ligands. However, little is known about the molecular mechanism of B3GNT3’s involvement in tumors and further study of this mechanism is required.

## Conclusions

In conclusion, this study is the first study evaluating the expression of B3GNT3 mRNA and protein in cervical cancer cell lines as well as early-stage cervical cancer specimens. Realtime-PCR and Western blotting analysis showed that mRNA and protein expression of B3GNT3 is upregulated in cervical cancer. Moreover, IHC and statistical analysis indicated that overexpression of B3GNT3 protein is correlated with HPV infection, FIGO stage, tumor size, tumor recurrence, vital status, concurrent chemotherapy and radiotherapy, lymphovascular space involvement and especially, lymph node metastasis. B3GNT3 protein levels could be used as an important prognostic marker of clinical outcome in early-stage cervical cancer patients.
